# Development of a qualitative assay for screening of *Bordetella pertussis* isolates for pertussis toxin production

**DOI:** 10.1371/journal.pone.0175326

**Published:** 2017-04-10

**Authors:** Irina Gates, Marina DuVall, Hong Ju, M. Lucia Tondella, Lucia Pawloski, for the Pertussis Working Group

**Affiliations:** Division of Bacterial Diseases, Centers for Disease Control and Prevention, Atlanta, Georgia, United States of America; Universidad Nacional de la Plata, ARGENTINA

## Abstract

*Bordetella pertussis* infection has been increasing in the US, with reported cases reaching over 50,000 in 2012, a number last observed in the 1950s. Concurrently, *B*. *pertussis* lacking the pertactin protein, one of the immunogens included in the acellular vaccine formulations, has rapidly emerged since 2010, and has become the predominant circulating phenotype. Monitoring the production of the remaining acellular vaccine immunogens, such as pertussis toxin (Pt), is a critical next step. To date, methods for screening Pt have been either through genomic sequencing means or by conventional ELISAs. However, sequencing limits detection to the DNA level, missing potential disruptions in transcription or translation. Conventional ELISAs are beneficial for detecting the protein; however, they can often suffer from poor sensitivity and specificity. Here we describe a rapid, highly sensitive and specific electrochemiluminescent capture ELISA that can detect Pt production in prepared inactivated bacterial suspensions. Over 340 isolates were analyzed and analytical validation parameters, such as precision, reproducibility, and stability, were rigorously tested. Intra-plate and inter-plate variability measured at 9.8% and 11.5%, respectively. Refrigerated samples remained stable for two months and variability was unaffected (coefficient of variation was 12%). Interestingly, despite the intention of being a qualitative method, the assay was sensitive enough to detect a small, but statistically significant, difference in protein production between different pertussis promoter allelic groups of strains, *ptxP1* and *ptxP3*. This technology has the ability to perform screening of multiple antigens at one time, thus, improving testing characteristics while minimizing costs, specimen volume, and testing time.

## Introduction

Pertussis, a highly contagious respiratory disease caused by the bacterium *Bordetella pertussis*, has made a resurgence globally among some highly vaccinated populations [1]. Epidemics have occurred in many US states, with national peaks in 2005, 2010, 2012 and 2014, reaching over 50,000 cases, and increasing endemicity throughout the US [2, 3]. Many reasons have been attributed to this resurgence, such as better diagnostics, more clinical awareness, and waning immunity from the acellular pertussis vaccine [4, 5].

It has also been postulated that the bacterium is genetically evolving in response to vaccine induced immunity, citing the major genetic shifts observed in the predominant allele types of the *B*. *pertussis* virulence factors that are included in the acellular vaccine formulations [6]. One such example is the genetic shift observed with the pertussis toxin promoter from *ptxP1* to *ptxP3* over the last few decades. An increase in the production of Pt has been associated with the genetic change to the *ptxP3* genotype, leading some to suggest a related increased virulence that could explain the global pertussis resurgence [7–11].

Most recently, there has been a dramatic increase in the loss of pertactin, one of the acellular vaccine components, among circulating strains in the US [12]. The loss of pertactin appears to be due to vaccine pressure among populations that are highly vaccinated with acellular pertussis vaccines [13]. Therefore, the question to be asked is if the bacterium will also respond to vaccine pressure with the loss of additional acellular vaccine components. Vaccine components in the two licensed vaccines in the US are pertussis toxin (Pt), filamentous hemagglutinin (Fha), and pertactin (Prn) with or without fimbriae 2 (Fim2) and 3 (Fim3). Other countries have identified the loss of other immunogens, albeit on very rare occasions and sporadically [14, 15]. In 2016, an isolate was reported to be both Prn- and Pt-deficient, the first of its kind in the US [16].

The loss of production of additional vaccine immunogens has been concerning for many countries that use only acellular pertussis vaccines. In response, many countries have in place, as part of their routine surveillance, testing for the production of vaccine immunogens by enzyme-linked immunosorbent assays (ELISA) [14]. Conventional ELISAs are simple, reproducible assays that can rapidly determine if a protein is being produced; however, they are also known to have small dynamic ranges and high background, which gives a narrow separation between positive and negative signals. Here we describe the development and validation of a new immunoassay for screening Pt production in circulating *B*. *pertussis* isolates, using electrochemiluminescence technology that provides more sensitivity, less background, requires much smaller volumes of reagents, and is highly reproducible, allowing for a much higher discrimination between positive and negative results.

## Materials and methods

### *Bordetella pertussis* isolates

Testing was performed on 346 *B*. *pertussis* isolates, consisting of 241 from the Enhanced Pertussis Surveillance (EPS), emerging infectious program sites CO, CT, GA, MN, NM, NY, and OR, from 2012–2015; 64 isolates from the 2010 and 2014 CA epidemics [3, 17]; 37 *ptxP1* isolates collected from 1947–2013; and four laboratory controls (Tohama I (two isolations), H921, and FR3749). The isolates were previously characterized in-house using a multi-locus sequence typing (MLST) scheme that includes the pertussis toxin operon and its promoter (*ptxA* and *ptxP*) as two of its targets [18]. Three isolates, FR3749, I979, and J365, do not produce Pt due to a 28kb deletion that includes the loss of the entire *ptx*/*ptl* operon, as confirmed by sequencing and western blot analyses [15, 16, 19]. H921 and purified Pt (Protein Express, Ohio, USA) were included as positive controls.

### Bacterial suspension inactivation

Several methods of bacterial inactivation were evaluated, which included treatment with disinfectants 5% MicroChem-Plus (National Chemical Laboratories, Pennsylvania, USA) and 20% chlorhexidine (MP Biomedicals, California, USA) for 5 minutes, UVC (254 nm) inactivation (time of inactivation included 15, 30, 45 minutes at 10 cm distance), freeze-thaw (three times), and bacterial xTractor lysis buffer (Clontech, California, USA) containing 25U of benzonase (Sigma-Aldrich, Missouri, USA). Inactivated bacterial cell suspensions from each treatment method were cultured on Regan-Lowe agar without cephalexin for 10 days at 37°C, and checked daily for microbial growth. Additionally, each inactivation method was evaluated on ease-of-use and throughput level. The lysis buffer was selected as the method for bacterial inactivation used during assay development.

### Bacterial cell culture and preparation

All *B*. *pertussis* isolates were stored frozen in -80°C at the first passage and sub-cultured on Regan-Lowe agar without cephalexin (prepared in-house) for 3 days at 37°C. Bacterial isolates were harvested and re-suspended in 2.0mL of in-house prepared 0.01M phosphate-buffered saline (PBS) to a final turbidity measurement of 0.95–1.05 using a Dade Behring MicroScan Turbidity Meter (Siemens Global, Munich, Germany). A 200μL aliquot of each suspension was transferred to a corresponding micro-centrifuge tube, and spun at 2655 x g for 10 minutes at room temperature in an Eppendorf benchtop centrifuge (model 5417C, rotor F45-30-11). To inactivate the cellular suspension, the bacterial pathogen supernatant was removed and the cell pellets were re-suspended in 200μL of xTractor lysis buffer (Clontech) containing 25U of benzonase (Sigma-Aldrich). Samples were vortexed for 10 minutes at room temperature, then centrifuged for an additional 10 minutes, at 2655 x g. Supernatants were removed and pellets were re-suspended in 200μL of 0.01M PBS. Prepared inactivated bacterial suspensions were stored at 4°C to be tested, in duplicate, the following day.

### Capture assay overview

This electrochemiluminescence (ECL)-based assay was developed on the SECTOR Imager 6000 platform (Meso-Scale Discovery, Maryland, USA), a high throughput ECL reader that detects emitted light from the non-radioactive label when it is electrochemically stimulated, producing minimal background signal. Cell-associated Pt was captured with 30uL/well of sheep anti-pertussis toxin serum (NIBSC, UK) solution prepared in PBS at 1:10,000 dilution, that was immobilized on high binding carbon electrodes surface of a standard MULTI-ARRAY 96-well plate (Meso-Scale Discovery, Maryland, USA) and incubated overnight at 4°C. The plate was washed on the following day with 0.05% PBS-tween wash buffer (Sigma-Aldrich) and blocked with 150μL of prepared blocking buffer for 30 minutes at room temperature on a shaking platform. The in-house prepared blocking buffer contained 2% goat serum (Sigma-Aldrich), 2% Blocker A (MSD) and 5% skim milk (Fisher Scientific, Pennsylvania, USA), diluted in PBS.

After blocking, the buffer was removed and 25μL of bacterial suspension was added to the plate and incubated on a shaking platform for one hour at room temperature. The plate was washed six times, with 300μL of wash buffer. Pt was detected with 25μL of mouse anti-pertussis toxin subunit 1 monoclonal antibody (NIBSC, UK, clone 10D6) at 1000-fold dilution prepared in blocking buffer [20, 21]. The plate was incubated on a shaking platform at room temperature for 45 minutes, then washed three times. Next, 25μL of goat anti-mouse sulfotag labeled antibody (MSD), prepared in blocking buffer at a 1μg/mL concentration, was added to the plate and incubated on a shaking platform at room temperature for 45 minutes. The plate was subsequently washed, 150μL of 2X Read Buffer T with surfactant (MSD) solution prepared in water was added to each well, and the plate was read immediately on the SECTOR Imager 6000. With the read buffer providing the appropriate chemical environment for ECL, when the plate is loaded into the instrument, a voltage is applied to the plate electrodes, causing the label bound to the electrode surface to emit light. The instrument measures the intensity of the emitted light to provide a quantitative measure of the amount of Pt present in the well.

Capture and detection antibody concentrations were optimized to minimize any non-specific binding to the negative control isolate which did not produce Pt. Signal that was produced by the negative control was comparable to background wells that contained PBS, instead of bacterial suspension.

### Data analysis

We used GraphPad Prism, version 6.0 (GraphPad, California, USA), to perform data analysis of results collected from SECTOR Imager 6000. A Mixed ANOVA Model was applied to the *ptxP1* vs. *ptxP3* comparison to analyze the difference in Pt production between the two groups, considering variations across plates and days.

### Assay specificity

To assess assay specificity, three additional *Bordetella* species that are known to not produce Pt, were tested for Pt production alongside the two *B*. *pertussis* Pt-negative isolates, I979 and J365. These included four isolates of *B*. *bronchiseptica*, five isolates of *B*. *holmesii*, and five isolates of *B*. *parapertussis*. Thirteen of the 14 isolates had a date of isolation known, which ranged from 2010–2015 and their states of origin included CT, MA, MN, and NY. The Pt-positive isolate, H921, and PBS were included as assay controls.

### Assay reproducibility

To evaluate assay reproducibility, the Z’-factor statistical method was applied, which is commonly used to estimate reproducibility and robustness of screening assays. This parameter assesses, in part, assay quality by calculating separation between positive and negative signals. Calculated Z’-values between 1 and 0.5 refer to an excellent assay, while values between 0.5 and 0 indicate a marginal assay. The Z’-factor was calculated using the following formula [22]:

$$Z-factor=1-\frac{3\times (\sigma \;PT\;positive+\sigma \;PT\;negative)}{\left(\mu \;PT\;positive-\mu \;PT\;negative\right)}$$

The Z-factor experiment was performed twice with positive and negative control isolates (H921 and FR3749, respectively) that were used throughout the assay development. In the first experiment, 80 positive and 16 negative control suspension replicates were tested. In the second experiment, 24 positive and 24 negative control isolates were tested.

### Assay variability

Inter-plate and intra-plate variability was evaluated on 20 *B*. *pertussis* isolates. Isolates were prepared as described above and stored at 4°C for the duration of the study. Each prepared isolate was tested on five different days, with 10 replicates per plate. Four replicates of positive control isolate, H921, and four replicates of negative control isolate, FR3749, were included on each plate. Coefficients of variation were calculated for both intra- and inter-plate variability. Inter-plate variability was calculated using normalized results for each isolate by expressing isolate signal values as a fraction of the averaged positive control signal given from their respective plate. For intra-plate variability, unadjusted signal values were used to calculate variability between replicates for each isolate on a plate.

### Sample stability

To determine sample stability post-lysis, a large batch of positive (H921) and negative (FR3749) isolate suspension controls were prepared and stored at 4°C for two months. Four replicates of each control isolate were included per plate and tested over a period of two months. In addition to the stability control suspension, freshly-prepared controls were also included, following the same number of passages as test isolates. For each control, the mean, standard deviation and coefficient of variation were calculated and the evaluated changes in signal strength over time were observed.

### Assay validation

During assay validation, 344 *B*. *pertussis* isolates were screened for Pt production. Samples were prepared as described above and each isolate was tested in duplicate on the same plate. Positive (H921) and negative (FR3749) control replicates were included on each plate. Replicate signal values were averaged for each isolate and control. In order to compare the results between experiments, isolate signal values were expressed as a fraction of the positive control reference signal value.

### *ptxP1* vs. *ptxP3* isolates comparison

The *B*. *pertussis* isolates used for this comparison consisted of 289 *ptxP3* and 50 *ptxP1* isolates that were randomly placed on the test plates. *PtxP3* isolates were made up of either EPS 2012–2015 or CA 2010 and 2014 epidemics, while *ptxP1* isolates ranged widely between years (1947–2013) and origin (18 states, the majority from MN (22%), CA (11%), and OR (11%)). Normalized signal values for both groups were used to compare the two populations for the differences in Pt signal levels. We applied T-test statistical analysis to determine if the two groups were different. In addition, a detailed comparison was performed of 10 *ptxP1* and 10 *ptxP3* that were collected in 2012 from MN. Only 10 *ptxP1* were received by MN for that year, therefore, the 10 *ptxP3* isolates were randomly selected from all those received in 2012. Five cellular preparations were made for each isolate and each preparation was run a total of five times (tested once/day) for a total of 25 measurements/isolate. The location of each preparation was randomly placed on the plate for each test.

## Results

### Inactivation of bacterial preparations

To comply with laboratory safety policies, several methods of bacterial inactivation were evaluated. Results of bacterial growth post-inactivation revealed that freeze-thaw and 15–30 minutes of UVC treatments were ineffective in inactivating the bacterial cells. UVC treatment for 45 minutes resulted in no bacterial growth, but was of very low throughput. No bacterial growth was also observed post-treatment with MicroChem-Plus, chlorhexidine and lysis buffer; therefore, cells inactivated by these methods were stained with a monoclonal antibody against Pt subunit 1 to verify epitope stability. Cell treatment with MicroChem-Plus and chlorhexidine lead to epitope destruction, with low to no signal; however, lysis buffer did not cause any changes in targeted epitope and produced a strong signal (data not shown). As a result, lysis buffer was selected as a method for bacterial inactivation. In order to improve assay signal-to-noise ratio, we evaluated the lysis buffer matrix effect and compared results collected from cells that were tested in lysis buffer to cells where lysis buffer was removed after lysis completion (data not shown). Removal of lysis buffer and re-suspension of cell pellet in PBS significantly improved signal-to noise ratio.

### Assay specificity

**Fig 1:**
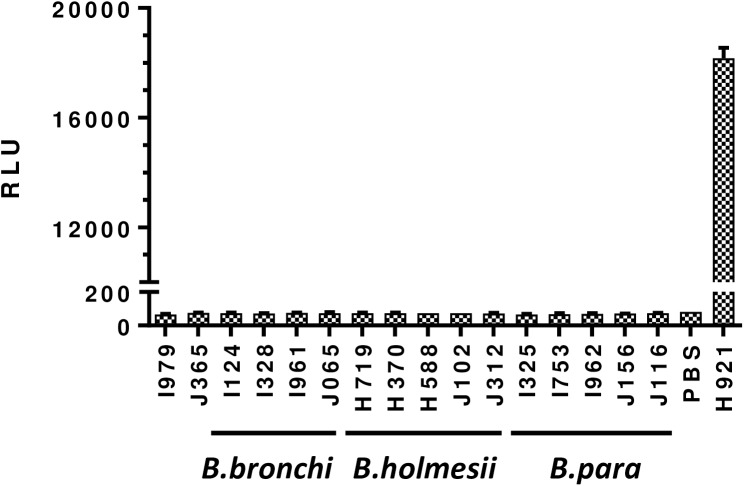
Assay specificity. *B*. *bronchiseptica* (*B*.*bronchi*), *B*. *holmesii*, and *B*. *parapertussis* (*B*.*para*) isolates (n = 14) were used to evaluate assay specificity, alongside *B*. *pertussis* Pt-negative isolates, I979 and J365. The H921 isolate is the *B*. *pertussis* Pt-positive isolate that was used as the positive control. Phosphate-buffered saline (PBS) was used as an additional assay control to screen for cross-reactivity. RLU = relative light units.

Fourteen isolates, consisting of *B*. *parapertussis*, *B*. *holmesii*, and *B*. *bronchiseptica* that are most closely related to *B*. *pertussis* but do not produce Pt, were evaluated alongside *B*. *pertussis* Pt-negative isolates (Fig 1 and S1 File). These *Bordetella* spp. isolates produced signals similar to PBS and Pt-negative isolates compared to the positive control that gave >200-fold difference in signal. These results confirm that the assay is highly specific for identifying Pt production.

### Assay reproducibility

To evaluate assay reproducibility, we applied the Z’-factor statistical method [22]. We calculated mean and standard deviations values for Pt positive and negative wells and used them to determine the Z’-factor value. The experiment was repeated and the averaged calculated Z’-factor of both experiments was 0.765 (standard deviation 0.021, percent coefficient of variation (%CV) was 2.77%), which falls within the range for a robust and reproducible assay.

### Assay variability

Inter-plate variability was evaluated on 20 isolates in five different experiments (Table 1). The analysis showed that mean %CV was 11.5% (min-7.6%, max-14.7%, median-11.3%). The calculated intra-plate variability was 9.79% (min-3.35%, max-20.1%, median-9.28%).

**Table 1:** Variability between runs*.

Isolate	Mean FOC	%CV
I125	**1.034**	**7.59**
I149	**1.118**	**10.47**
I315	**1.087**	**9.30**
I327	**1.121**	**12.74**
I337	**1.015**	**10.10**
I338	**0.828**	**10.00**
I339	**0.817**	**14.71**
I340	**0.817**	**14.71**
I342	**1.027**	**9.82**
I343	**1.056**	**11.45**
I344	**0.766**	**12.85**
I347	**1.226**	**10.51**
I348	**1.046**	**10.63**
I359	**1.107**	**12.27**
I430	**1.012**	**12.36**
I434	**1.111**	**11.05**
I437	**1.227**	**9.72**
I746	**0.751**	**14.57**
I747	**0.956**	**13.94**
I748	**1.005**	**11.66**

*Assay variability between runs was evaluated on 20 *B*. *pertussis* isolates. Isolates were prepared as described above and stored at 4°C for the duration of the study. Each prepared isolate was tested on 5 different days, with 10 replicates per plate. Four replicates of positive control isolate (H921) was included on each plate. To normalize results for each isolate we averaged signal values for positive control on each plate and expressed isolate signal values as a fraction of a positive control signal. FOC = fraction of control.

### Sample stability post-lysis and storage at 4°C

Signal stability over time was performed on a batch of positive and negative controls in a period of two months. Inactivated bacterial suspensions were stored at 4°C and included in experiments over a period of two months. We did not observe any changes in signal strength and the calculated %CV was 12%.

### Assay validation

**Fig 2:**
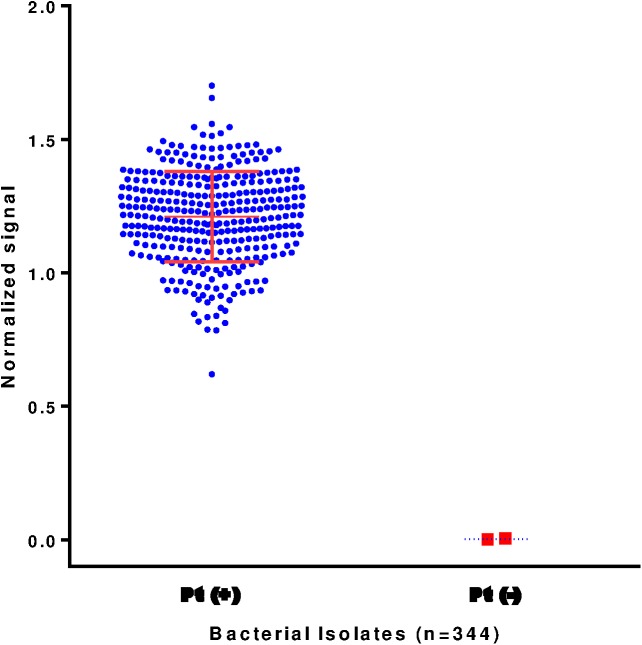
Assay validation. *B*. *pertussis* (n = 344) isolates were screened for pertussis toxin (Pt) presence. Signal values for each isolate were normalized to their corresponding positive control and expressed as a fraction of the positive control value. The two Pt-deficient isolates, I979 and J365, were identified as Pt-negative by the assay. Pt-positive control had a ratio = 1, while Pt-negative had a ratio = 0.

During assay validation, we screened 344 *B*. *pertussis* isolates (Fig 2 and S1 File). Out of 344 screened bacterial suspensions, only the two previously identified Pt-negative isolates (I979 and J365) were identified as negative for Pt production. The identification of isolate I979 and J365 as Pt-negative correlated with previous molecular sequencing results, western blot analysis, and genomic sequencing [15, 16]. Overall, the majority of isolates had Pt signal higher than selected positive control isolate (81.2%), and 8.7% isolates had signal lower than positive control. Thirty out of 344 isolates (8.7%) had signal similar to the selected positive control.

### *ptxP1* vs. *ptxP3* comparison

**Fig 3:**
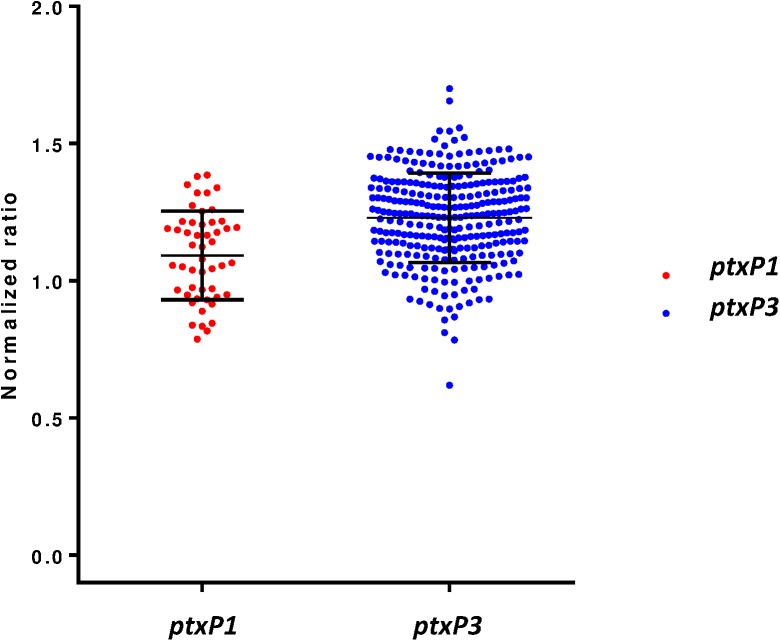
Comparison of Pt signal level between 50 *ptxP1* and 289 *ptxP3 B*. *pertussis* isolates. Mean normalized signal values were 1.093 (SEM ±0.02286, n = 50) and 1.230 (SEM ±0.009601) for *ptxP1* and *ptxP3* isolates, respectively (p value < 0.0001).

We included a set of 50 *ptxP1* isolates to be compared with 289 *ptxP3* isolates for assessing a possible difference in Pt production levels (Fig 3 and S1 File). Normalized signal values for two groups were compared using unpaired two-tailed t-test analysis. Results showed that the two groups differed significantly with respect to Pt production (p value < 0.0001). Mean normalized signal value for *ptxP3* isolates were 1.230 (SEM ±0.009601, n = 289) and for *ptxP1* were 1.093 (SEM ±0.02286, n = 50).

To remove any potential bias as to year and origin of isolation, as well as any potential testing (cell preparation, plate, and day) bias, an additional experiment to compare a set of 10 *ptxP1* and 10 *ptxP3* isolates, from the same year and location, was performed. Multiple cellular preparations on multiple plates and days were tested and evaluated through a Mixed ANOVA Model used for analyzing the variance. The difference in mean signal values remained similar to the initial analysis (1.10 and 0.92 for *ptxP3* and *ptxP1* isolates, respectively) and that difference was still statistically significant (F = 49.52, P = 0.003) when considering day and plate as random effects. The results also indicated that the variance between days and plates was not statistically significant (P = 0.16 and P = 0.25, respectively).

## Discussion

Through PCR and western blot analysis, circulating *B*. *pertussis* isolates have been identified to have recently gone through rapid, drastic genetic changes to eliminate the production of pertactin, one of the five immunogens that are included in the acellular pertussis vaccine [12]. This loss was likely a result of vaccine pressure from the first US cohort to have been solely vaccinated with the acellular vaccine as their primary series [13]. The question arose whether other vaccine antigens were also undergoing the same pressure. Recently, two double immunogen-deficient (Pt and Prn) isolates have been reported [16, 19].

Immunoassays are a convenient and inexpensive method for rapidly screening bacterial isolates for the production of vaccine antigens [14]. However, conventional ELISA-based assays require large sample volumes and are known to have high background and low dynamic range. In contrast, electrochemiluminescence-based immunoassays offer a more sensitive method, with little to no non-specific binding observed and broad dynamic range. In this paper, we describe the development and validation of a new Pt-screening immunoassay using the MSD platform. During assay development, two previously characterized *B*. *pertussis* isolates as positive (H921) and negative (FR3749) controls were used, based on their molecular characterization [12, 15]. During method development, the optimized assay conditions resulted in at least a 600-fold signal difference between positive and negative controls. Signal values from negative control were similar to the PBS background.

For assay validation, 344 *B*. *pertussis* isolates that were previously characterized by MLST [18, 23] were screened. The screening identified the two Pt-deficient isolates (I979 and J365) as Pt-negative, while all other isolates were Pt-positive. This finding correlates with MLST characterization. The signal level associated with Pt protein varied between isolates, and after normalization to positive control, 81.9% of isolates had Pt signal somewhat higher than positive control isolate (1.1–1.7 fold difference), and 9.3% of isolates had slightly lower levels of protein production (0.9–0.6 fold difference), showing that the selected positive control may lean more towards the lower end of the signal range. Although, the developed method was not intended to quantify Pt level production by different isolates, the variance in Pt-associated signal was noted.

In the Netherlands, a shift in the predominant MLST type for the *ptxP* allele from *ptxP1* to *ptxP3* was first observed in the mid-1990s alongside a significant increase in pertussis notifications; additionally, the authors observed a small, but statistically significant, difference in the *in vitro* production of Pt [11]. This shift from the genotype *ptxP1* to *ptxP3* has now been observed worldwide, including the US [23–25]. Interestingly, while this immunoassay was not meant to quantify Pt production differences, our results from multiple analyses did demonstrate that this immunoassay was sensitive enough to detect a slightly higher, statistically significant, level of Pt production from *ptxP3* isolates than *ptxP1* isolates.

The ECL technology on the MSD platform proved to be a reliable, sensitive, and specific method for the screening of *B*. *pertussis* isolates for pertussis toxin production. This technology allows screening of multiple targets in the same sample, which provides significant savings in cost and time. Future work includes the development of assay conditions for detection of other *B*. *pertussis* pathogenic factors, like pertactin, filamentous hemagglutinin, and fimbriae 2 and 3, which will be multiplexed together in a new screening *B*. *pertussis* immunoassay.

## Supporting information

S1 File Supporting information. Excel file of source data.
(XLSX)
